# Avoidable Mortality Among People with Disabilities in South Korea: A Nationwide 10-Year Trend Analysis, 2015–2024

**DOI:** 10.3390/healthcare14213617

**Published:** 2026-10-26

**Authors:** Ye-Soon Kim, Mi Jin Kim, Seung Hee Ho, Seon-Deok Eun

**Affiliations:** Department of Healthcare and Public Health Research, Rehabilitation Research Institute, National Rehabilitation Center, Seoul 01022, Republic of Korea; yesoon@korea.kr (Y.-S.K.); mijinkimhealth@korea.kr (M.J.K.); hsh7@korea.kr (S.H.H.)

**Keywords:** avoidable mortality, preventable mortality, treatable mortality

## Abstract

**Background:** People with disabilities experience health inequities and premature mortality, but evidence on changes in avoidable mortality remains limited. Monitoring preventable and treatable mortality can help describe changes in premature deaths that may be amenable to public health measures or healthcare. **Objectives:** This study examined trends in avoidable, preventable, and treatable mortality among registered people with disabilities in South Korea from 2015 to 2024 and assessed differences by disability characteristics. **Methods:** We analyzed linked data from the Registered Disability Database and Cause of Death Statistics. Causes of death were classified according to the OECD/Eurostat criteria, and age-standardized mortality rates were calculated using the 2005 total population as the standard population. Annual percentage changes (APCs) were estimated using a log-linear regression model. **Results:** Overall age-standardized avoidable mortality decreased by 5.68% over a 10-year period (APC, −0.1%; 95% CI, −2.1 to 1.9). Preventable mortality decreased by 11.74% (APC, −0.6%; 95% CI, −2.8 to 1.7), whereas treatable mortality increased by 4.07% (APC, 0.7%; 95% CI, −0.9 to 2.3). None of the overall mortality trends was statistically significant. However, treatable mortality increased significantly among adults aged 45–64 years (APC, 2.1%; p < 0.05) and people with mild disabilities (APC, 2.0%; p < 0.05). Treatable mortality was high among people with developmental and brain lesion disabilities; cerebrovascular disease, renal failure, and aspiration pneumonia were major causes of death. **Conclusions:** These findings support continued monitoring of avoidable mortality and further investigation into factors potentially associated with mortality patterns across disability groups, including healthcare access and utilization. Such evidence may inform disability-inclusive health policies.

## 1. Introduction

People with disabilities consistently report a lower health status and a higher risk of premature mortality than people without disabilities [1,2]. The World Health Organization (WHO) highlights that people with disabilities often die at younger ages and experience worse health; these health inequities stem not only from the disability itself but also from social, environmental, and healthcare system barriers [3,4]. To mitigate these health inequities, continuous monitoring of health outcomes and the identification of actionable health issues through prevention and healthcare services are essential [3,5]. As an objectively measurable health outcome, mortality provides an important indicator for assessing population health and health system performance, identifying health inequalities, and informing policy priorities for intervention [6,7].

However, the overall mortality rates alone cannot capture the extent of premature deaths, which can be reduced through prevention or adequate healthcare services. Avoidable mortality addresses this gap, defining premature deaths that could be prevented or treated through effective, timely public health and healthcare interventions [8,9,10,11]. Avoidable mortality is broadly categorized into preventable and treatable mortality, allowing population-level premature deaths to be evaluated from two distinct policy perspectives: public health prevention and healthcare delivery [9,11]. Globally, avoidable mortality levels and trends serve as key indicators for assessing healthcare system performance and setting public health priorities [12,13].

To enhance international comparability, the OECD and Eurostat developed and regularly update a shared classification framework for preventable and treatable mortality [14]. This standardized framework is widely used to compare the scale and trends of avoidable mortality across countries, supporting long-term evaluations of the overall mortality reduction and structural shifts in the causes of death [12,13,14]. Consequently, avoidable mortality provides a useful public health indicator for assessing the potential contribution of preventive interventions and timely, effective healthcare to reducing premature deaths, complementing overall mortality rates and enabling the monitoring of temporal trends [3,15].

Although studies on avoidable mortality among people with disabilities remain relatively limited compared to those on the general population, evidence is steadily accumulating. Research from a nationwide Danish cohort demonstrated a substantial burden of avoidable mortality among adults with intellectual disabilities, with elevated risks of both preventable and treatable mortality compared with the general population, particularly for treatable mortality [16]. Furthermore, a recent systematic review identified pneumonia, aspiration pneumonia, epilepsy, cerebrovascular disease, ischemic heart disease, deep vein thrombosis, diabetes mellitus, and sepsis as the major potential causes of avoidable death in people with intellectual disabilities [17]. These findings underscore the importance of examining premature mortality among people with disabilities as a public health concern, given that some health disparities may arise from avoidable social disadvantages and deficiencies in preventive and healthcare services, rather than from the underlying disability alone [11,18].

Previous studies in South Korea have examined avoidable mortality among people with disabilities. Using the National Health Insurance cohort data from 2003 to 2013, Choi et al. demonstrated that people with disabilities experienced significantly higher levels of both preventable and treatable mortality than those without disabilities [19]. Although this provided foundational domestic evidence of mortality inequality from an avoidable mortality perspective, more than a decade has passed since the study period. Recent Korean literature on avoidable mortality has predominantly focused on general or regional populations. Recent Korean studies have reported declining long-term trends in avoidable mortality and regional variations in avoidable mortality [20,21]. Consequently, nationwide evidence evaluating recent long-term trends and temporal changes in avoidable mortality, specifically among Korean people with disabilities, remains scarce.

Therefore, using the most recent cause-of-death data available up to 2024, this study aimed to analyze the 10-year trends (2015–2024) in avoidable mortality among people with disabilities in South Korea. By examining temporal changes across overall avoidable, preventable, and treatable mortality, as well as shifts in major causes of death, this study quantified the scale and trajectory of reducible premature deaths. Ultimately, this study provides empirical evidence to support long-term mortality monitoring and to guide the development of future disability-inclusive health policies.

## 2. Materials and Methods

### 2.1. Data Sources

This study used nationwide linked data from the disability registration database maintained by the Korea Social Security Information Service and cause-of-death data maintained by Statistics Korea for the period from 2015 to 2024. In South Korea, persons with disabilities are managed through a national disability registration system, and Statistics Korea maintains cause-of-death data collected on the basis of death certificates and related documentation. The two datasets were linked by Statistics Korea through its MicroData Integrated Service (MDIS) using personal identifiers. Before the linked data were provided to the research team, all personally identifiable information was removed, and the records were randomly reordered. According to the data-linkage agency, no cases were omitted during the linkage process. Causes of death were classified according to the 8th revision of the Korean Standard Classification of Diseases (KCD-8) [22]. The annual registered population with disabilities was defined as the population as of 31 December of each year.

This study was approved by the Institutional Review Board of the National Rehabilitation Center (NRC-2026-01-001).

### 2.2. Registered People with Disabilities

The disability registration system in South Korea operates under the Act on Welfare of Persons with Disabilities, which manages individuals who meet the statutory criteria for disability types and severity as registered people with disabilities [23]. The study population was defined as individuals registered in the official disability registration system in Korea. Registered people with disabilities were categorized into 15 statutory disability types based on the classification system in the registry data: physical, brain lesions, visual, hearing, speech, facial, renal, cardiac, liver, respiratory, ostomy/urostomy, epilepsy, intellectual, autistic, and mental disabilities.

Disability severity was defined considering the policy reforms that occurred during the study period. Before July 2019, disabilities were classified into grades 1 through 6; thereafter, the grading system was abolished and simplified into a two-tier system: “severe disability” and “mild disability” [24]. In this study, the mortality patterns according to severity were analyzed using the disability severity information provided in the dataset.

### 2.3. Definition and Classification of Avoidable Mortality

Avoidable mortality was defined on the basis of the 2022 criteria of the OECD/Eurostat Lists of Preventable and Treatable Causes of Death [14]. Preventable and treatable death counts are weighted and presented to one decimal place. Some deaths are included in both categories but counted once in total avoidable mortality; therefore, the sum of these counts may exceed the total avoidable death count. This classification system focuses on premature deaths among individuals aged <75 years and categorizes avoidable mortality into preventable and treatable mortality. Preventable mortality refers to deaths that can be prevented through effective public health and primary prevention measures, whereas treatable mortality refers to deaths that can be avoided through timely and effective healthcare interventions after the onset of a disease or injury [14].

In this study, the KCD-8 cause-of-death codes were mapped based on the causes of death and the International Statistical Classification of Diseases 10th Edition (ICD-10) codes listed in the OECD/Eurostat [14] framework. The corresponding KCD-8 codes were selected following a review of the definitions and inclusion scope for each cause of death in the OECD/Eurostat list, establishing a finalized mapping list applicable to the Korean national cause-of-death statistics. The final mapped causes of death, OECD/Eurostat classifications, ICD-10 codes, and KCD-8 codes are shown in Table S1.

### 2.4. Statistical Analyses

The annual registered population with disabilities and the number of deaths were calculated along with the avoidable, preventable, and treatable mortality rates. Mortality rates were calculated by using the registered population with disabilities for each year as the denominator. To account for changes in the age structure of people with disabilities during the study period, age-standardized mortality rates (ASMRs) were calculated by direct standardization using the 2005 total population as the standard population. In accordance with the OECD/Eurostat framework, the analysis was restricted to deaths at ages under 75 years [14]. Age-specific mortality rates were calculated using deaths and the corresponding mid-year registered populations with disabilities in the following age groups (age 0, ages 1–4, and five-year intervals from ages 5–9 through 70–74). These rates were multiplied by the corresponding standard population weights and summed to obtain ASMRs per 100,000 populations. The original weights for ages 0–74, which sum to 0.971545777, were retained without renormalization to sum to 1, so that the estimates represent the contribution of deaths at ages under 75 to the mortality rate standardized to the full population.

To evaluate the temporal trends in avoidable mortality across the study years, the annual percentage change (APC) was calculated. APCs were estimated for overall avoidable mortality, as well as for preventable and treatable mortality separately. Furthermore, trend analyses were performed for major causes of death, sex, and disability type. APCs and their corresponding 95% confidence intervals (CIs) are presented for all trends. We assessed residual normality, model specification, and autocorrelation, and compared linear and quadratic models using the significance of the quadratic term and the corrected Akaike information criterion (AICc). The log-linear model was retained to provide a consistent summary of the average annual percent change (APC) over the entire study period across mortality categories. The APCs were estimated using log-linear regression models, with the natural logarithm of the annual ASMR as the dependent variable. Study years were coded as a continuous variable starting from 0 in 2015, and the APC was calculated from the estimated regression coefficient (β) as [(exp(β) − 1) × 100]. The three leading causes of death were identified separately for preventable and treatable mortality within each disability type, based on pooled ASMRs for 2015–2024. For each disability type, cause of death, and age group, weighted deaths were summed across the study period and divided by the sum of the corresponding annual mid-year populations. These pooled age-specific rates were standardized as described above. The resulting pooled ASMRs were used to rank causes of death.

All statistical analyses were conducted using SAS version 9.4 (SAS Institute Inc., Cary, NC, USA). Statistical significance was evaluated using two-tailed tests at p < 0.05, with p < 0.10 representing marginal significance.

## 3. Results

### 3.1. General Study Population Characteristics

The baseline characteristics of the registered population with disabilities in 2015, 2019, and 2024 are presented in Table 1. The total registered population with disabilities increased from 2,486,595 in 2015 to 2,631,348 in 2024, while the sex distribution remained stable throughout the study period, at approximately 58% male and 42% female.

Regarding age structure, a distinct aging trend was observed. The mean age increased from 59.0 to 63.5 years. The proportion of individuals aged ≥75 years increased by 11.6 percentage points (from 20.2% to 31.8%), whereas that of individuals aged 45–64 years decreased by 9.1 percentage points (from 39.0% to 29.9%). Regarding disability severity, the proportion of individuals with severe disabilities decreased from 38.7% to 36.7%, whereas that of those with mild disabilities increased from 61.3% to 63.3%.

With respect to disability type, the proportion of individuals with physical disabilities decreased from 51.5% to 43.0%, whereas that of individuals with hearing disabilities increased from 10.1% to 16.8%. In addition, the proportion of individuals with renal, intellectual, and autistic disabilities showed an increasing trend.

### 3.2. Trends in Avoidable Mortality Among People with Disabilities: 2015–2024

From 2015 to 2024, the age-standardized avoidable mortality rate among registered people with disabilities showed an overall declining trend; however, no statistically significant change was observed (Table 2). The avoidable mortality rate decreased by 5.68%, from 417.5 per 100,000 population in 2015 to 393.8 per 100,000 population in 2024, with an APC of −0.1% (95% CI, −2.1 to 1.9; p > 0.05).

The preventable mortality rate decreased by 11.74%, from 238.8 per 100,000 population in 2015 to 210.7 per 100,000 population in 2024, with an APC of −0.6% (95% CI, −2.8 to 1.7), which was not statistically significant (p = 0.583). Conversely, the treatable mortality rate increased by 4.07% during the same period, from 187.1 per 100,000 population to 194.7 per 100,000 population, with an APC of 0.7% (95% CI, −0.9 to 2.3), showing no statistically significant upward trend (p > 0.05).

Annual patterns revealed that avoidable mortality rates generally declined from 2015 to 2021, experienced a temporary spike to 452.6 per 100,000 population in 2022, and subsequently decreased to 391.8 per 100,000 population in 2023 and 393.8 per 100,000 population in 2024. Similar temporal fluctuations were observed for preventable and treatable mortality rates.

### 3.3. Trends in Avoidable Mortality by Disability Characteristics: 2015–2024

An analysis of the trends in avoidable mortality rates by sex, age, disability severity, and disability type revealed distinct variations across the subgroups (Table 3). By sex, no statistically significant changes in avoidable mortality rates were observed for either males (APC, −0.6%; 95% CI, −2.4 to 1.2) or females (APC, 0.3%; 95% CI, −2.3 to 3.0). By age, treatable mortality rates increased significantly among individuals aged 45–64 years (APC, 2.1%; 95% CI, 0.6 to 3.5), with an increasing trend also observed in the 20–44 years age group (APC, 1.7%; 95% CI, −0.3 to 3.6). Among those aged 65–74 years, no statistically significant trends were observed across any of the three mortality indicators.

Regarding disability severity, no significant changes in avoidable mortality rates were observed among individuals with severe disabilities. Similarly, overall avoidable mortality rates among individuals with mild disabilities showed no significant change; however, their treatable mortality rates increased significantly (APC, 2.0%; 95% CI, 0.1 to 3.9), whereas their preventable mortality rates exhibited a declining trend (APC, −1.3%; 95% CI, −2.6 to 0.0).

By disability type, statistically significant decreases in avoidable mortality rates (APC, −2.3%; 95% CI, −3.6 to −1.0) and preventable mortality rates (APC, −2.9%; 95% CI, −3.9 to −1.9) were observed among individuals with visual disabilities. Significant reductions were observed in liver disabilities for both avoidable mortality (APC, −4.9%; 95% CI, −9.0 to −0.7) and preventable mortality (APC, −6.4%; 95% CI, −10.7 to −1.9). In cardiac disabilities, treatable mortality rates decreased significantly (APC, −6.6%; 95% CI, −11.9 to −0.8). Conversely, no statistically significant changes in avoidable mortality rates were observed among individuals with physical disabilities, brain lesions, intellectual disabilities, or autistic disabilities.

### 3.4. Top Three Major Causes of Death by Disability Type

**Figure 1:**
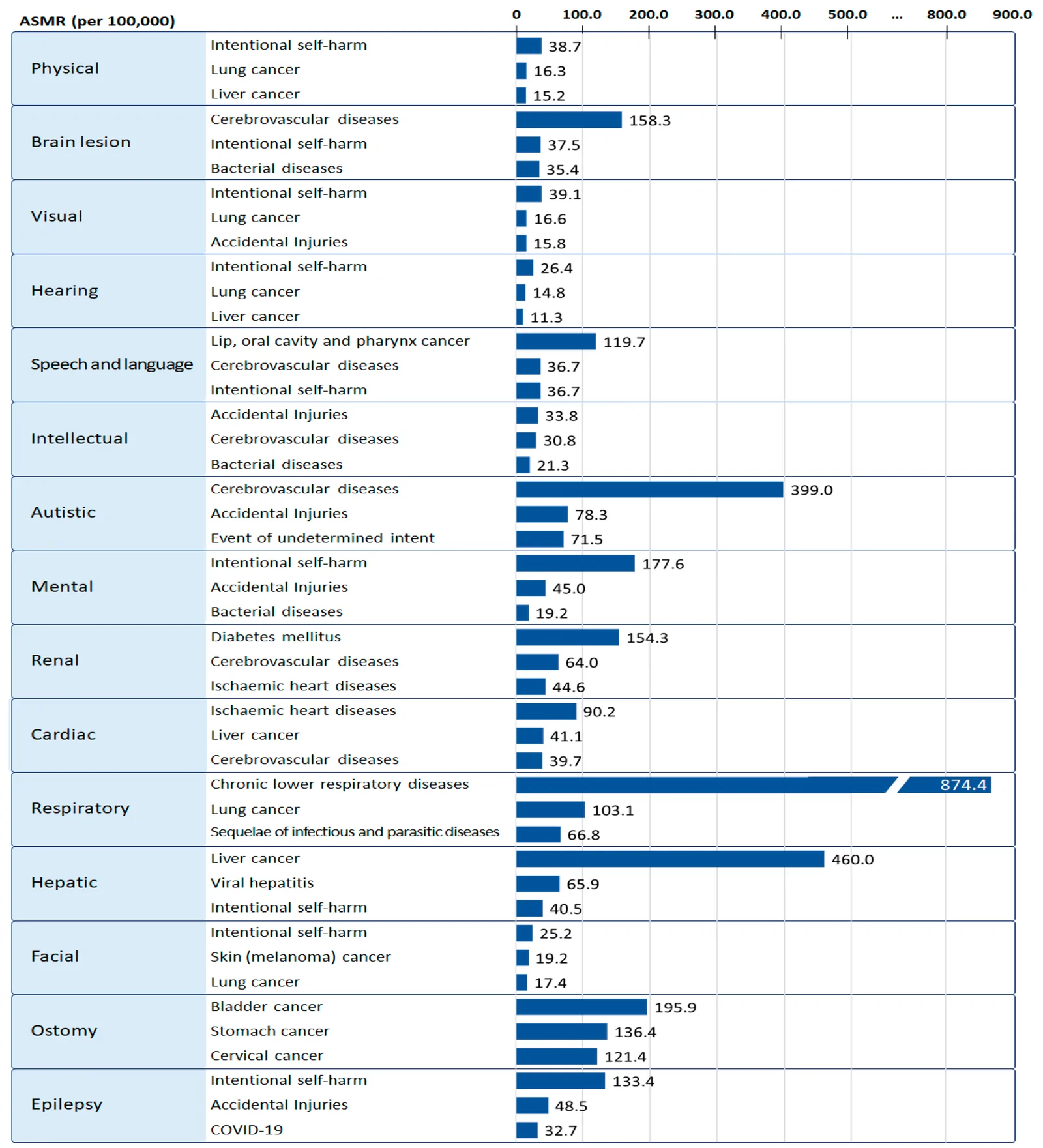
Top three causes of preventable deaths by disability type, 2015–2024.

**Figure 2:**
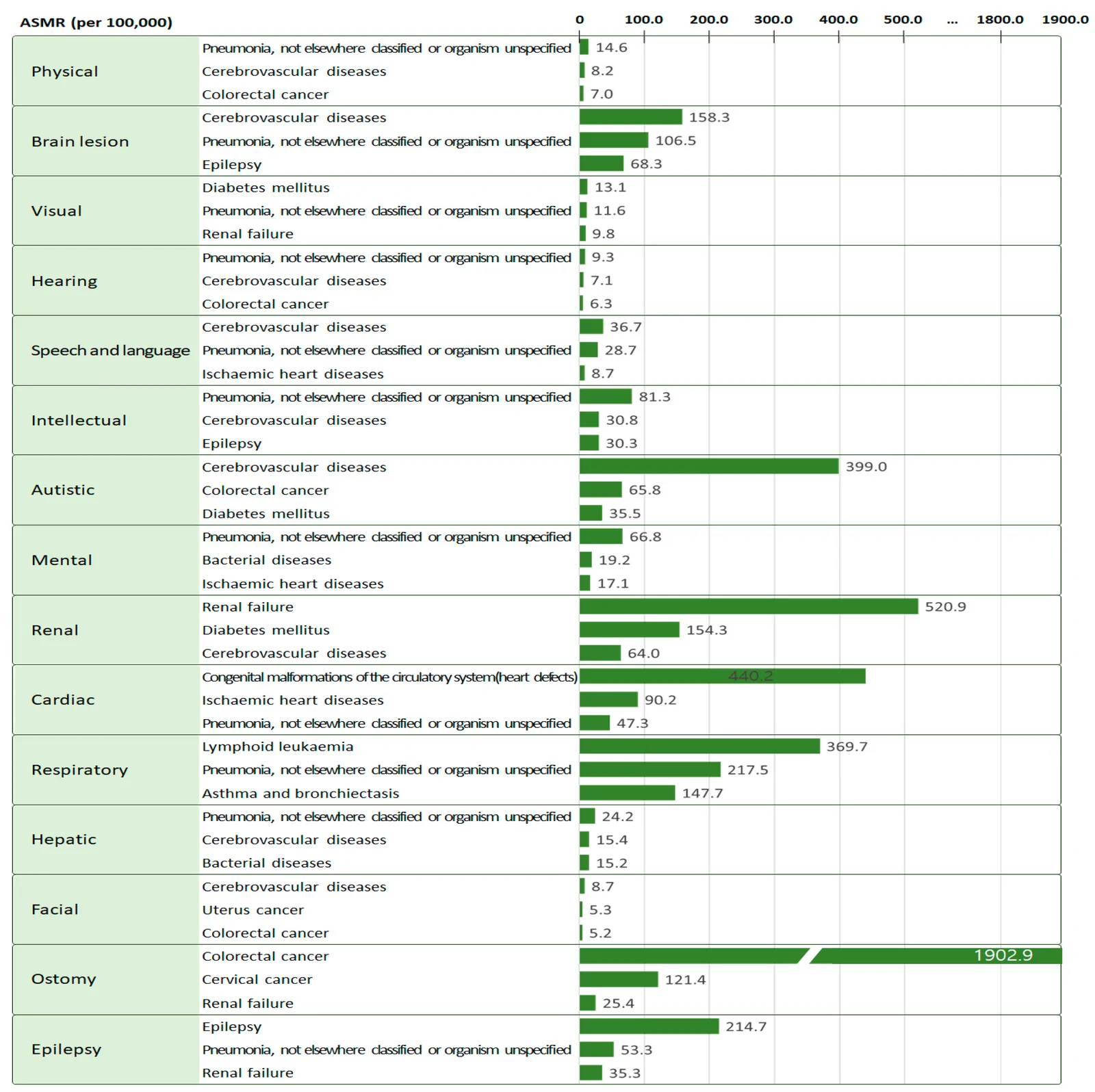
Top three causes of treatable deaths by disability type, 2015–2024.

A comparison of the top three preventable and treatable causes of death by disability type from 2015 to 2024 revealed distinct variations across the disability categories (Figure 1 and Figure 2). Intentional self-harm was the leading cause of death among individuals with physical, visual, hearing, mental, and facial disabilities. Cerebrovascular disease was the leading cause among individuals with brain-lesion and autistic disabilities, while accidental injuries ranked highest among those with intellectual disabilities. Among the internal organ disabilities, the predominant causes of death were diabetes mellitus for renal disabilities, ischemic heart disease for cardiac disabilities, chronic lower respiratory diseases for respiratory disabilities, and liver cancer for liver disabilities.

Distinct variations in disability types were also identified in the treatable causes of death (Figure 2). Cerebrovascular disease was the leading treatable cause of death among individuals with brain-lesion disabilities (158.3 per 100,000 population), renal failure among those with renal disabilities (520.9 per 100,000 population), and congenital malformations of the circulatory system among those with cardiac disabilities (440.2 per 100,000 population). Furthermore, colorectal cancer was the leading treatable cause of death among individuals with ostomy/urostomy disabilities (1902.9 per 100,000 population), whereas epilepsy was the leading cause among those with epilepsy disabilities (214.7 per 100,000 population).

## 4. Discussion

This study comprehensively analyzed long-term trends and subgroup-specific levels of avoidable, preventable, and treatable mortality among registered people with disabilities in South Korea from 2015 to 2024 using nationwide linked big data combining health and welfare registries with national cause-of-death statistics.

Although the overall age-standardized avoidable mortality rate among people with disabilities decreased by approximately 5.68% over the past decade, this change was not statistically significant (APC, −0.1%). Preventable mortality, which is closely tied to public health and primary prevention interventions, declined by 11.74% (APC, −0.6%). In contrast, treatable mortality, which can be reduced through timely and effective healthcare interventions, increased by 4.07% (APC, 0.7%). However, none of the three overall mortality indicators showed a statistically significant temporal trend during the study period This differs from previous evidence from the general Korean population, in which declines in avoidable mortality were largely attributable to reductions in preventable mortality [19]. Although the observed patterns differed between preventable and treatable mortality, this study did not directly assess the effects of public health interventions or healthcare-related factors. Therefore, the extent to which these factors may have contributed to the observed mortality patterns remains unclear [3,19].

The observed decrease in preventable mortality may be consistent with broader changes in population-level prevention and public health in Korea [20,24]. In contrast, treatable mortality increased over the study period, although the overall trend was not statistically significant. This finding does not establish the presence or effect of systemic healthcare inequities. Subgroup analyses revealed a statistically significant increase in treatable mortality among working-age adults aged 45–64 years (APC, 2.1%; p < 0.05) and individuals with mild disabilities (APC, 2.0%; p < 0.05). These findings may reflect persistent barriers to timely and appropriate healthcare among people with disabilities [3,19,25]. Previous research has documented disparities in healthcare access and utilization among people with disabilities, including lower participation in preventive health screening and poorer accessibility of preventive or outpatient healthcare services [26,27]. However, the present study did not directly assess healthcare access or utilization; therefore, the extent to which these disparities contributed to the observed mortality patterns remains uncertain.

Our findings also demonstrated a disproportionate burden of avoidable mortality across disability types and severities. Statistically significant declines in avoidable and preventable mortality were observed among people with visual (APC, −2.3%) and liver disabilities (APC, −4.9%), while treatable mortality decreased significantly among those with cardiac disabilities. In contrast, no statistically significant changes in overall avoidable mortality were observed among people with physical, brain lesion, intellectual, and autistic disabilities. Previous research has documented challenges in healthcare access and the recognition of health needs among people with developmental disabilities, including intellectual and autistic disabilities [16,17,26,27].

However, the present study did not directly assess healthcare access, diagnostic delays, or treatment processes; therefore, these factors cannot be established as explanations for the mortality patterns observed. Cause-specific analyses further revealed differences across disability types: cerebrovascular disease was a leading treatable cause of death among people with brain-lesion disabilities, renal failure among those with renal disabilities, and epilepsy among those with epilepsy disabilities. These findings underscore the importance of examining mortality patterns by disability type and investigating whether differences in healthcare access, utilization, and the management of condition-specific health needs are associated with these patterns.

Analysis of the top causes of death by disability type revealed that, alongside external factors such as suicide (intentional self-harm) and traffic accidents, conditions arising from physical vulnerabilities specific to certain disability types emerged as important contributors to treatable mortality [17]. However, current primary care and rehabilitation systems may lack adequate home-based care options such as home-visit rehabilitation, dysphagia therapy, and respiratory care for evaluating and managing complications at home [3].

Although this study yielded meaningful insights by analyzing avoidable mortality among registered people with disabilities in South Korea, several limitations should be noted. First, the study population was restricted to registered individuals with disabilities and did not include unregistered individuals. In South Korea, disability registration rates vary considerably according to disability type and severity, which may have limited the generalizability of the findings to all individuals with disabilities. Future studies should seek to include unregistered individuals with disabilities through data linking with relevant health and disability records.

Second, owing to the population-level design, individual-level confounding variables could not be adjusted for. Factors such as income, education, health behaviors, comorbidities, geographic region of residence, and healthcare utilization were closely associated with both disability severity and avoidable mortality; however, these variables were not available in the dataset. Consequently, it remains unclear whether the observed disparities across disability types and severities reflect the direct effect of the disability itself or are attributable to unmeasured socioeconomic factors. Future research utilizing a triple-linked cohort, combining disability registry data, health insurance claims data, and cause-of-death statistics, is required to establish individual-level causal relationships. In addition, the study did not directly measure healthcare access, healthcare utilization, or exposure to public health interventions. Accordingly, the observed mortality patterns cannot be attributed to these factors, and their potential contributions should be examined in future studies using linked healthcare utilization and clinical data.

Third, the classification of avoidable mortality relies solely on underlying causes of death. By relying exclusively on the underlying cause of death, disability-contributing deaths involving multiple causes (e.g., brain lesion disability → swallowing dysfunction → aspiration pneumonia → death) may have been missed, potentially underestimating treatable mortality. Future studies should complement these findings by conducting sensitivity analyses using multiple cause-of-death data to enhance the validity.

Fourth, the COVID-19 pandemic may have influenced avoidable mortality trends during the study period, particularly the observed peak in 2022. However, we retained COVID-19 deaths in the primary analysis in accordance with the OECD/Eurostat classification of avoidable mortality, which categorizes COVID-19 as a preventable cause of death. Therefore, the extent to which COVID-19 deaths influenced the estimated long-term trends could not be separately assessed in this study. Future research should further examine mortality trends using complementary analyses to distinguish the contribution of COVID-19 deaths from trends in other avoidable causes.

Despite these limitations, this study is the first to identify structural stagnation in treatable mortality among people with disabilities using a decade of nationwide data. Based on these findings, several policy and clinical recommendations are proposed.

To address the stagnation in treatable mortality and health disparities across disability types identified in this study, the following policies and clinical interventions are needed [3,19,24,27]. First, primary care physician programs and home-visit medical services for people with disabilities should be activated. For individuals with severe disabilities who have mobility limitations or developmental disabilities and face challenges in visiting medical institutions, the fee schedules and incentive structures of the primary care physician system should be reformed to enable healthcare providers to conduct home visits for chronic disease management and early detection of complications [3,24,27]. Second, a comprehensive system for the prevention of complications and primary rehabilitation tailored to specific disability characteristics should be established. Standardized manuals for dysphagia management, respiratory care, and infection control education should be disseminated through primary care facilities and community welfare centers for individuals with brain lesions or neurological disabilities [3,17]. Third, disability-inclusive emergency transportation and medical delivery systems should be strengthened. Collaborative networks between emergency transport services and regional emergency medical centers could be considered to facilitate information sharing regarding disability characteristics and timely emergency care. Fourth, integrated health, medical, and welfare community care should be established. Beyond basic social support, a fully integrated community care system is needed to monitor health status, manage medication adherence, and ensure routine health checkup compliance among people with disabilities [3,27].

## 5. Conclusions

This study analyzed trends in age-standardized avoidable mortality rates among registered people with disabilities in South Korea over a 10-year period (2015–2024). Although preventable mortality decreased by 11.74% and treatable mortality increased by 4.07%, none of the three overall mortality trends was statistically significant. However, treatable mortality increased significantly in specific subgroups, particularly among working-age adults aged 45–64 years and individuals with mild disabilities. The persistent burden of high-risk causes of death in specific disability groups, including those with intellectual, autistic, and brain lesion disabilities, further highlights the need to investigate factors potentially associated with these mortality patterns.

These findings support continued monitoring of avoidable mortality and further investigation into factors potentially associated with mortality patterns across disability groups, including healthcare access and utilization. Such evidence may inform disability-inclusive health policies aimed at reducing avoidable deaths and improving health equity. Policy efforts could include strengthening primary care physician programs and home-visit medical and rehabilitation services, establishing disability-inclusive emergency medical networks, and integrating health, medical, and welfare services in community care.

## Figures and Tables

**Table 1:** Characteristics of the registered disability population in South Korea, 2015–2024.

Characteristics	2015 n (%)	2019 n (%)	2024 n (%)	Absolute Change in Proportion, 2015–2024 (%p)
Total Registered Population	2,486,595 (100.0)	2,614,344 (100.0)	2,631,348 (100.0)	
Sex				
Male	1,444,802 (58.1)	1,510,385 (57.8)	1,525,050 (58.0)	−0.1
Female	1,041,793 (41.9)	1,103,959 (42.2)	1,106,298 (42.0)	+0.1
Age Group				
0–19 yr	89,561 (3.6)	90,740 (3.5)	102,766 (3.9)	+0.3
20–44 yr	376,382 (15.1)	327,972 (12.5)	286,963 (10.9)	−4.2
45–64 yr	968,999 (39.0)	935,132 (35.8)	785,841 (29.9)	−9.1
65–74 yr	550,064 (22.1)	556,715 (21.3)	618,506 (23.5)	+1.4
≥75 yr	501,589 (20.2)	703,785 (26.9)	837,272 (31.8)	+11.6
Mean Age (M ± SD), yr	59.0 ± 17.7	61.4 ± 18.3	63.5 ± 19.0	
Disability Severity ^1^				
Severe (Grade 1–3/Severe)	963,267 (38.7)	982,094 (37.6)	966,424 (36.7)	−2.0
Mild (Grade 4–6/Mild)	1,523,328 (61.3)	1,632,250 (62.4)	1,664,924 (63.3)	+2.0
Major Disability Types				
Physical	1,280,141 (51.5)	1,221,871 (46.7)	1,132,639 (43.0)	−8.4
Brain lesion	250,144 (10.1)	250,935 (9.6)	234,703 (8.9)	−1.1
Visual	252,576 (10.2)	252,688 (9.7)	246,182 (9.4)	−0.8
Hearing	249,903 (10.1)	376,561 (14.4)	442,033 (16.8)	+6.7
Speech and language	18,774 (0.8)	21,431 (0.8)	22,389 (0.9)	+0.1
Facial	2684 (0.1)	2670 (0.1)	2756 (0.1)	+0.0
Renal	74,117 (3.0)	91,798 (3.5)	111,084 (4.2)	+1.2
Cardiac	5809 (0.2)	5221 (0.2)	4781 (0.2)	−0.1
Liver	10,291 (0.4)	13,112 (0.5)	16,098 (0.6)	+0.2
Respiratory	11,969 (0.5)	11,403 (0.4)	10,512 (0.4)	−0.1
Ostomy	14,066 (0.6)	15,230 (0.6)	17,397 (0.7)	+0.1
Epilepsy	7048 (0.3)	7047 (0.3)	6889 (0.3)	+0.0
Intellectual	189,566 (7.6)	212,827 (8.1)	233,320 (8.9)	+1.2
Autistic	21,083 (0.8)	28,669 (1.1)	47,350 (1.8)	+1.0
Mental	98,424 (4.0)	102,881 (3.9)	103,215 (3.9)	−0.1

^1^ Disability severities were reclassified in July 2019: Grades 1–3 were classified as severe and Grades 4–6 as mild. Pre-2019 data were mapped accordingly. Note: Values are presented as n (%). Change represents the absolute difference in population proportion between 2024 and 2015, calculated as the 2024 proportion minus the 2015 proportion, and is expressed in percentage points (%p). Source: Korea Social Security Information Service. Population as of 31 December of each year. M, mean; SD, standard deviation.

**Table 2:** Trends in age-standardized mortality rates of avoidable, preventable, and treatable mortality among registered persons with disabilities, 2015–2024.

Year	Total Avoidable Mortality		Preventable Mortality		Treatable Mortality	
	ASR (95% CI)	Deaths (n)	ASR (95% CI)	Deaths (n)	ASR (95% CI)	Deaths (n)
2015	417.5 (405.9–429.2)	20,413.0	238.8 (231.5–246.0)	12,033.5	187.1 (178.1–196.0)	8719.5
2016	417.9 (406.4–429.4)	20,107.0	238.5 (231.0–246.0)	11,740.0	188.7 (180.2–197.3)	8792.0
2017	376.9 (366.0–387.9)	18,012.0	212.5 (205.5–219.5)	10,418.0	174.9 (166.7–183.1)	8051.0
2018	375.2 (364.4–386.1)	18,009.0	212.7 (205.4–220.0)	10,294.0	174.2 (166.5–181.8)	8202.0
2019	368.7 (358.1–379.3)	17,545.0	206.9 (199.9–213.9)	10,043.0	173.0 (165.2–180.8)	7977.0
2020	359.9 (349.6–370.3)	17,333.0	209.1 (201.5–216.8)	9921.0	165.5 (158.4–172.6)	8005.0
2021	359.8 (349.6–370.1)	17,861.0	200.3 (193.8–206.8)	10,225.0	173.5 (165.9–181.1)	8300.0
2022	452.6 (440.2–464.9)	21,557.0	264.3 (256.2–272.5)	13,056.0	200.7 (191.9–209.6)	9145.0
2023	391.8 (380.4–403.3)	18,753.0	214.9 (207.6–222.2)	10,550.0	192.3 (183.8–200.8)	8926.0
2024	393.8 (382.8–404.8)	18,437.0	210.7 (203.8–217.7)	10,128.5	194.7 (186.7–202.7)	8869.5
APC (95% CI)	−0.1 (−2.1–1.9)		−0.6 (−2.8–1.7)		0.7 (−0.9–2.3)	
p-value	0.887		0.583		0.363	
Overall Change (%)	−5.68%		−11.74%		4.07%	

ASR, age-standardized rate per 100,000 persons, standardized to the 2005 total population; APC, annual percentage change estimated using log-linear regression; CI, confidence interval. Note 1: 2020–2022 includes the COVID-19 pandemic period. Note 2: Preventable and treatable death counts are weighted and presented to one decimal place. Some deaths are included in both categories but counted once in total avoidable mortality; therefore, the sum of these counts may exceed the total avoidable death count.

**Table 3:** Trends in avoidable mortality by characteristics among registered persons with disabilities, 2015–2024.

Subgroup	Avoidable Mortality APC (95% CI)	Preventable Mortality APC (95% CI)	Treatable Mortality APC (95% CI)
Total (Overall)	−0.1 (−2.1, 1.9)	−0.6 (−2.8, 1.7)	0.7 (−0.9, 2.3)
Sex			
Male	−0.6 (−2.4, 1.2)	−1.4 (−3.3, 0.6)	0.7 (−0.9, 2.4)
Female	0.3 (−2.3, 3.0)	1.0 (−2.3, 4.3)	0.1 (−1.9, 2.1)
Age Group			
20–44 yr	0.7 (−1.4, 2.8)	0.1 (−2.2, 2.5)	1.7 (−0.3, 3.6)
45–64 yr	0.4 (−1.5, 2.3)	−0.6 (−2.8, 1.6)	2.1 (0.6, 3.5) *
65–74 yr	−0.7 (−2.6, 1.2)	−0.8 (−3.3, 1.7)	−0.2 (−1.4, 1.0)
Disability Severity			
Severe (Grade 1–3/Severe)	−0.3 (−2.5, 1.9)	−0.6 (−3.4, 2.3)	0.2 (−1.3, 1.8)
Mild (Grade 4–6/Mild)	−0.3 (−1.8, 1.2)	−1.3 (−2.6, 0.0)	2.0 (0.1, 3.9) *
Major Disability Types			
Physical	−0.6 (−2.9, 1.8)	−1.2 (−3.3, 1.0)	0.6 (−2.8, 4.2)
Brain lesion	0.8 (−2.0, 3.7)	0.9 (−2.7, 4.6)	1.2 (−0.8, 3.3)
Visual	−2.3 (−3.6, −1.0) **	−2.9 (−3.9, −1.9) ***	−1.2 (−5.4, 3.2)
Hearing	−1.7 (−3.3, 0.0)	−1.8 (−3.7, 0.3)	−1.4 (−3.9, 1.0)
Speech and language	−1.0 (−3.8, 2.0)	−0.5 (−3.7, 2.7)	−1.4 (−6.1, 3.5)
Facial	−5.4 (−12.2, 1.8)	−7.9 (−15.8, 0.8)	2.0 (−5.1, 9.8)
Renal	−1.5 (−6.8, 4.0)	0.6 (−3.5, 4.8)	−2.1 (−8.3, 4.5)
Cardiac	−4.5 (−9.9, 1.3)	0.7 (−8.8, 11.1)	−6.6 (−11.9, −0.8) *
Liver	−4.9 (−9.0, −0.7) *	−6.4 (−10.7, −1.9) *	8.9 (−3.3, 22.7)
Respiratory	−4.1 (−8.4, 0.5)	−1.1 (−5.3, 3.2)	−7.0 (−14.3, 0.9)
Ostomy	−3.7 (−8.3, 1.2)	−2.5 (−8.7, 4.3)	−3.8 (−9.4, 2.1)
Epilepsy	0.6 (−5.5, 7.1)	−2.9 (−12.2, 7.4)	3.2 (−7.1, 14.6)
Intellectual	−0.3 (−2.5, 2.0)	−0.6 (−3.7, 2.7)	0.2 (−1.4, 1.9)
Autistic	16.5 (−25.3, 81.7)	17.7 (−25.8, 86.8)	16.2 (−28.3, 88.3)
Mental	0.0 (−4.8, 5.1)	−0.2 (−6.3, 6.2)	0.3 (−1.9, 2.6)

APC annual percentage change; CI, confidence interval. Log-linear regression of annual age-standardized mortality rates was used to estimate APCs. *** p < 0.001; ** p < 0.01; * p < 0.05. APC estimates indicate no statistically significant increase or decline in mortality (95% CI includes 0; α = 0.05). Disability severity was reclassified in July 2019: grades 1–3 → “Severe”; grades 4–6 → “Mild”. Pre-2019 data were mapped accordingly.

## Data Availability

There are limitations to the availability of this data. The data was provided by Statistics Korea and is available through the MicroData Integrated Service (MDIS) with permission from Statistics Korea. Further inquiries can be directed to the corresponding author.

## Supplementary Materials

The following supporting information can be downloaded at: https://www.mdpi.com/article/10.3390/healthcare14213617/s1, Table S1. Mapping of Causes of Avoidable Mortality Defined by OECD/Eurostat to KCD-8 Codes.

## Abbreviations

The following abbreviations are used in this manuscript:

APC	Annual percentage change
ASMR	Age-standardized mortality rate
KCD	Korean Standard Classification of Diseases
NRC	National Rehabilitation Center
ICD-10	International Statistical Classification of Diseases 10th Edition
ASR	Age-standardized rate

## References

[1] Kuper H., Rotenberg S., Azizatunnisa’ L., Banks L.M., Smythe T. (2024). The association between disability and mortality: A mixed-methods study. Lancet Public Health.

[2] Smythe T., Rotenberg S., Moyo-Chilufya M., Wilbur J., Kuper H. (2025). Health inequalities among people with disabilities: An umbrella review and evidence synthesis. eClinicalMedicine.

[3] World Health Organization (2022). Global Report on Health Equity for Persons with Disabilities.

[4] World Health Organization (2011). World Report on Disability.

[5] World Health Organization (2023). Disability and Health.

[6] Liang C.Y., Kornas K., Bornbaum C., Shuldiner J., De Prophetis E., Buajitti E., Pach B., Rosella L.C. (2023). Mortality-based indicators for measuring health system performance and population health in high-income countries: A systematic review. IJQHC Commun..

[7] Pan American Health Organization (2021). Health in the Americas 2021: Potentially Avoidable Premature Mortality. https://hia.paho.org/en/avoidable-mortality.

[8] Rutstein D.D., Berenberg W., Chalmers T.C., Child C.G., Fishman A.P., Perrin E.B. (1976). Measuring the quality of medical care: A clinical method. N. Engl. J. Med..

[9] Nolte E., McKee M. (2003). Measuring the health of nations: Analysis of mortality amenable to health care. BMJ.

[10] Nolte E., McKee M. (2004). Does Health Care Save Lives? Avoidable Mortality Revisited.

[11] Nolte E., McKee C.M. (2008). Measuring the health of nations: Updating an earlier analysis. Health Aff..

[12] Nolte E., McKee M. (2011). Variations in amenable mortality—Trends in 16 high-income nations. Health Policy.

[13] Gay J.G., Paris V., Devaux M., de Looper M. (2011). Mortality Amenable to Health Care in 31 OECD Countries: Estimates and Methodological Issues.

[14] OECD/Eurostat (2022). Avoidable Mortality: OECD/Eurostat Lists of Preventable and Treatable Causes of Death (January 2022 Version).

[15] Meade M.A., Mahmoudi E., Lee S.Y. (2015). The intersection of disability and healthcare disparities: A conceptual framework. Disabil. Rehabil..

[16] Thygesen L.C., Klitgaard M.B., Sabers A., Kjellberg J., Søndergaard J., Sørensen J., Sonne M., Juel K., Michelsen S.I. (2024). Potentially avoidable mortality among adults with intellectual disability. Eur. J. Public Health.

[17] Heslop P., Lauer E. (2024). Strategies to prevent or reduce inequalities in specific avoidable causes of death for adults with intellectual disability: A systematic review. Br. J. Learn. Disabil..

[18] Organisation for Economic Co-operation and Development (2023). Health at a Glance 2023: OECD Indicators.

[19] Choi M.H., Kim A.R., Yoon T.H. (2018). Health inequality between people with and without disability and impact factors. Korean Data Anal. Soc..

[20] Jeong Y., Seo S. (2024). Avoidable mortality in Korea 1997–2021: Temporal trend and its contribution to all-cause mortality. Int. J. Public Health.

[21] Yang J.M., Hwang J. (2024). Incidence and influencing factors of avoidable mortality in Korea from 2013–2022: Analysis of cause-of-death statistics. J. Prev. Med. Public Health.

[22] Korean Standard Classification of Diseases, 8th Revision (KCD-8). https://www.koicd.kr/kcd/kcd.do.

[23] Republic of Korea Act on Welfare of Persons with Disabilities.

[24] Ministry of Health and Welfare (2019). Policy for Persons with Disabilities: User-Centric Disability Support System.

[25] Krahn G.L., Walker D.K., Correa-De-Araujo R. (2015). Persons with disabilities as an unrecognized health disparity population. Am. J. Public Health.

[26] Iezzoni L.I. (2011). Eliminating health and health care disparities among the growing population of people with disabilities. Health Aff..

[27] Jeon B., Kwon S., Kim H. (2015). Health care utilization by people with disabilities: A longitudinal analysis of the Korea Welfare Panel Study (KoWePS). Disabil. Health J..

