# Supplementary material for: Avoidable Mortality Among People with Disabilities in South Korea: A Nationwide 10-Year Trend Analysis, 2015–2024
Source: Healthcare (Basel). 2026 Oct 26;14(21):3617. doi: 10.3390/healthcare14213617 (PMC13763422; doi:10.3390/healthcare14213617)
Supplement: Supplementary file 1 [file healthcare-14-03617-s001.zip › healthcare-4604823-supplementary.pdf]

Table S1: Mapping of Causes of Avoidable Mortality Defined by OECD/Eurostat to KCD-8 Codes.

Causes of deaths	Avoidable mortality	ICD-10 Code	KCD-8 Code	Notes (Korean Context)
**Infectious diseases**				
Intestinal Diseases	Preventable	A00–A09	A00–A09	
Infections with a predominantly sexual mode of transmission	Preventable	A50-A64	A50-A64	
Viral infections characterized by skin and mucous membrane lesions	Preventable	B00-B09	B00-B09	
Viral hepatitis	Preventable	B15-B19	B15-B19	HBV vaccination / perinatal prevention
HIV/AIDS	Preventable	B20-B24	B20-B24	National HIV/AIDS prevention and care
Protozoal diseases	Preventable	B50-B64	B50-B64	
Bacterial diseases	100:100	A30-A49	A30-A49	
Bacterial meningitis, NEC	100:100	G00	G00	
Meningitis due to other and unspecified causes	Treatable	G03	G03	
Tuberculosis	50:50	A15–A19	A15–A19	National TB control program
Sequelae of infectious and parasitic diseases	50:50	B90-B94	B90-B94	
Pneumoconiosis associated with tuberculosis	50:50	J65	J65	
**Cancer**				
Lip, oral cavity and pharynx cancer	Preventable	C00–C14	C00–C14	
Oesophageal cancer	Preventable	C15	C15	
Stomach cancer	Preventable	C16	C16	National cancer screening
Liver cancer	Preventable	C22	C22	HBV prevention / national screening
Lung cancer	Preventable	C33–C34	C33–C34	Smoking control / national screening
Mesothelioma	Preventable	C45	C45	
Skin (melanoma) cancer	Preventable	C43	C43	
Bladder cancer	Preventable	C67	C67	
Colorectal cancer	Treatable	C18–C21	C18–C21	National cancer screening
Breast cancer	Treatable	C50	C50	National cancer screening
Uterus cancer	Treatable	C54, C55	C54, C55	
Malignant neoplasm of testis	Treatable	C62	C62	
Malignant neoplasm of thyroid gland	Treatable	C73	C73	
Hodgkin lymphoma	Treatable	C81	C81	
Lymphoid leukaemia	Treatable	C91	C91	

Benign neoplasms	Treatable	D10–D36	D10–D36	
Cervical cancer	50:50	C53	C53	HPV vaccination / national screening
**Endocrine and metabolic diseases**				
Nutritional deficiency anaemia	Preventable	D50-D53	D50-D53	
Thyroid disorders	Treatable	E00-E07	E00-E07	
Adrenal disorders	Treatable	E24, E25, E27	E24, E25, E27	
Diabetes mellitus	50:50	E10-E14	E10-E14	National screening and chronic disease management
**Diseases of the nervous system**				
Epilepsy	Treatable	G40–G41	G40–G41	
**Diseases of the circulatory system**				
Rheumatic and other heart disease	Treatable	I00-I02, I05-I09	I00-I02, I05-I09	
Venous thromboembolism	Treatable	I26, I80, I82	I26, I80, I82	
Hypertensive diseases	50:50	I10-I13, I15	I10-I13, I15	National screening and chronic disease management
Ischaemic heart diseases	50:50	I20-I25	I20-I25	
Cerebrovascular diseases	50:50	I60-I69	I60-I69	
Other atherosclerosis	50:50	I70, I73	I70, I73	
Aortic aneurysm and dissection	50:50	I71	I71	
**Diseases of the respiratory system**				
Influenza	Preventable	J09-J11	J09-J11	National vaccination program
Pneumonia due to Streptococcus pneumonia or Haemophilus influenza	Preventable	J13-J14	J13-J14	National vaccination program
Chronic lower respiratory diseases	Preventable	J40-J44	J40-J44	Tobacco control / air pollution management
Lung diseases due to external agents	Preventable	J60-J64, J66-J70, J82, J92	J60-J64, J66-J70, J82, J92	Occupational/environmental exposure control
Upper respiratory infections	Treatable	J00-J06, J30-J39	J00-J06, J30-J39	
Pneumonia, not elsewhere classified or organism unspecified	Treatable	J12, J15, J16-J18	J12, J15, J16-J18	
Acute lower respiratory infections	Treatable	J20–J22	J20–J22	
Asthma and bronchiectasis	Treatable	J45-J47	J45-J47	
Adult respiratory distress syndrome	Treatable	J80	J80	
Pulmonary oedema	Treatable	J81	J81	
Abscess of lung and mediastinum pyothorax	Treatable	J85-J86	J85-J86	

Other pleural disorders	Treatable	J90, J93, J94	J90, J93, J94	
**Diseases of the digestive system**				
Gastric and duodenal ulcer	Treatable	K25-K28	K25-K28	
Appendicitis	Treatable	K35-K38	K35-K38	
Abdominal hernia	Treatable	K40-K46	K40-K46	
Cholelithiasis and cholecystitis	Treatable	K80-K81	K80-K81	
Other diseases of gallbladder or biliary tract	Treatable	K82-K83	K82-K83	
Acute pancreatitis	Treatable	K85	K85	
Other diseases of pancreas	Treatable	K86	K86	
**Diseases of the genitourinary system**				
Nephritis and nephrosis	Treatable	N00-N07	N00-N07	
Obstructive uropathy	Treatable	N13, N20-N21, N35	N13, N20-N21, N35	
Renal failure	Treatable	N17-N19	N17-N19	
Renal colic	Treatable	N23	N23	
Disorders resulting from renal tubular dysfunction	Treatable	N25	N25	
Unspecified contracted kidney, small kidney of unknown cause	Treatable	N26-N27	N26-N27	
Inflammatory diseases of genitourinary system	Treatable	N34, N70-N73, N75, N76	N34, N70-N73, N75, N76	
Hyperplasia of prostate	Treatable	N40	N40	
**Pregnancy, childbirth and perinatal period**				
Pregnancy, childbirth and the puerperium	Treatable	O00–O99	O00–O99	Maternal health care
Certain conditions originating in the perinatal period	Treatable	P00–P96	P00–P96	Perinatal and neonatal care
**Congenital malformations**				
Certain congenital malformations (neural tube defects)	Preventable	Q00, Q01, Q05	Q00, Q01, Q05	Prenatal care / folic acid supplementation support
Congenital malformations of the circulatory system (heart defects)	Treatable	Q20-Q28	Q20-Q28	
**Adverse effects of medical and surgical care**				
Drugs, medicaments and biological substances causing adverse effects in therapeutic use	Treatable	Y40–Y59	Y40–Y59	Adverse drug reaction relief system
Misadventures to patients during surgical and medical care	Treatable	Y60–Y69, Y83–Y84	Y60–Y69, Y83–Y84	Medical device adverse event reporting system
Medical devices associated with adverse incidents in diagnostic and therapeutic use	Treatable	Y70–Y82	Y70–Y82	

**Injuries**				
Transport Accidents	Preventable	V01-V99	V01-V99	Road traffic fatality reduction measures
Accidental Injuries	Preventable	W00-X59	W00-X59	Occupational and workplace safety / public and disaster safety policies
Intentional self-harm	Preventable	X60-X84	X60-X84	National suicide prevention policy
Assault	Preventable	X85-Y09	X85-Y09	
Event of undetermined intent	Preventable	Y10-Y34	Y10-Y34	
**Provisional assignment of new diseases**				
COVID-19	Preventable	U07, U00-U19	U07, U00-U19	Vaccination and infectious disease response

Note: Allocation weights (preventable : treatable) are 1:0 for exclusively preventable causes, 0:1 for exclusively treatable causes, 0.5:0.5 for "50:50," and 1:1 for "100:100." The 50:50 allocations follows the OECD/Eurostat framework. "100:100" represents a study-specific adaptation for two aggregated categories: Bacterial diseases and Bacterial meningitis, NEC. Limited coding detail in the supplied data prevented separation of preventable and treatable deaths within these categories; consequently, deaths were included in both categories. Each death is counted once in total avoidable mortality.
